# Anthropomorphic Design in Mortality Salience Situations: Exploring Emotional and Non-Emotional Mechanisms Enhancing Consumer Purchase Intentions

**DOI:** 10.3390/bs14111041

**Published:** 2024-11-05

**Authors:** Cong Sun, Yuechun Ding, Xinyi Wang, Xing Meng

**Affiliations:** 1 Pan Tianshou College of Architecture, Art and Design, Ningbo University, Ningbo 315211, China; suncong@nbu.edu.cn (C.S.); 2211370004@nbu.edu.cn (Y.D.); 2211370012@nbu.edu.cn (X.W.); 2 School of Business, Ningbo University, Ningbo 315211, China

**Keywords:** anthropomorphic design, mortality salience, consumer emotions, death anxiety, purchasing behavior, emotion regulation, psychological closeness, terror management theory

## Abstract

This study investigates the role of anthropomorphic design in alleviating consumer anxiety induced by mortality salience and delves into the underlying emotional and non-emotional mechanisms. Through a series of meticulously designed experiments, we confirm that anthropomorphic design significantly enhances positive emotional responses in consumers, reduces negative emotions, and thereby increases their preference and willingness to purchase products. Even after the diminution of emotional reactions, anthropomorphic design continues to sustain consumer preference by enhancing psychological intimacy. These findings reveal the crucial role of anthropomorphic design as an effective emotional regulation strategy in consumer purchasing behavior, enriching the application of terror management theory and emotion regulation theory in consumer behavior research. Furthermore, our study provides valuable practical guidance for product design and marketing strategies, especially for consumer groups frequently facing high-pressure situations. Products with anthropomorphic designs may be more appealing to these consumers, helping to mitigate their death anxiety and enhance psychological well-being.

## 1. Introduction

In everyday consumer behavior, emotions play a pivotal role in driving consumer purchasing and consumption decisions [1]. Specifically, the human cognition of death—mortality salience—as a potent emotional state significantly impacts individual purchasing behavior and product value perception [2]. As consumer behavior evolves, purchasing is increasingly seen not just as a means to fulfill material needs but as a vital pathway for emotional expression and identity affirmation [3]. Therefore, understanding how emotional responses drive purchasing decisions in contexts of mortality salience is crucial for insights into the interplay between emotion regulation, psychological well-being, and consumer behavior.

Existing research has demonstrated that mortality salience can trigger anxiety and discomfort, thereby influencing consumer choices [4,5]. Consumers often engage in purchasing behaviors to alleviate this death-related anxiety, seeking psychological safety and a sense of belonging. This not only acts as an emotional regulation strategy but also as a crucial mechanism to counteract death-related anxieties [6]. While terror management theory has explored the relationship between emotion regulation and mortality salience, the role of anthropomorphic design as an emotional regulation strategy within consumer environments has not been thoroughly examined.

Anthropomorphic design, the attribution of human characteristics to inanimate objects, can foster emotional connections, enhancing consumers’ sense of belonging and security [7]. This study proposes that in contexts of mortality salience, anthropomorphic design not only increases consumer purchase desire but also helps mitigate negative emotional responses associated with death anxiety through emotional regulation mechanisms. This theoretical framework applies terror management theory to consumer behavior research, offering a new perspective on the emotional regulatory functions of anthropomorphic designs in consumer environments.

The primary objective of this research is to explore the potential role of anthropomorphic designs in alleviating the negative emotional consequences induced by mortality salience. Through multiple experiments, we aim to reveal how anthropomorphic products influence individual emotional responses and purchasing behavior in the face of death reminders. Notably, as an innovative strategy, anthropomorphic design has been widely applied in products and services, establishing profound emotional bonds and promoting intimate consumer–product relationships [7,8,9,10,11,12].

Although previous research has explored various emotional regulation strategies, such as mindfulness and cognitive behavioral therapy, in mitigating death anxiety [13,14,15], anthropomorphic design stands out as an external intervention. Its uniqueness lies in its ability to reduce individual psychological burdens in a subtle yet effective manner by endowing products with human-like characteristics [7,11]. Unlike strategies that focus on internal emotional regulation (e.g., self-talk, physical exercise) [16], anthropomorphic design offers a new type of emotional buffer, particularly suitable for high-stress professions such as firefighters, healthcare workers, and police officers [17].

Existing emotional regulation strategies, like mindfulness and cognitive behavioral therapy, are effective but largely rely on an individual’s active engagement. In contrast, anthropomorphic design automatically elicits positive emotional responses through changes in the external environment, namely, the anthropomorphic elements in product design, thus alleviating death anxiety. This strategy is not only universal but also effective without requiring active effort from individuals. Therefore, the role of anthropomorphic design in contexts of mortality salience offers a new perspective for understanding emotional regulation in consumers, filling a gap in existing research.

At the theoretical level, this study significantly expands the research scope of the role of anthropomorphic design in mortality salience contexts. It deepens our understanding of anthropomorphic design’s emotional regulation functions and validates its positive impacts at non-emotional levels [18,19]. Through meticulous empirical research, this study has clarified the positive effects of anthropomorphic design on key dimensions such as sense of control, meaning in life, psychological closeness, and sense of belonging [20,21], thus strongly advancing the research and ongoing development in this field.

In summary, this study, by exploring the role of anthropomorphic design in emotional regulation, further reveals how emotions influence purchasing and consumption decisions, especially in contexts of death anxiety. Anthropomorphic design is not only an aesthetic choice but also a design strategy that triggers emotional responses in consumers, serving to alleviate emotional stress, enhance psychological well-being, and increase product appeal and market value in specific consumer environments. The findings of this study hold significant practical implications for brands, designers, and marketers, and also pave new pathways for research into the complex relationship between emotions and consumer behavior within the field of consumer psychology.

The structure of this paper is as follows: Section 2 establishes a theoretical framework, discussing how anthropomorphic design can alleviate anxiety brought on by mortality salience through terror management theory (TMT) and emotional regulation mechanisms. Section 3 and Section 4 examine the effects of anthropomorphic design on consumer preferences and emotional states in mortality salience contexts, through experiments with functional and entertainment products, respectively. Section 5 further explores the durability of these effects over time and the mechanisms behind them. Finally, Section 6 summarizes the research findings, discusses their theoretical and practical significance, and proposes directions for future research.

## 2. Theoretical Framework and Hypotheses

This study integrates terror management theory (TMT), emotion regulation theory, and anthropomorphism. TMT posits that the awareness of one’s own mortality can trigger existential anxiety, compelling individuals to seek cultural worldviews and bolster self-esteem [22]. Emotion regulation theory explores how individuals manage and adjust their emotional experiences [23]. In contexts of mortality salience, anthropomorphism—the attribution of human characteristics to non-human entities—is seen as a novel emotional regulation mechanism.

This research synthesizes these concepts and proposes the hypothesis that everyday items with anthropomorphic designs can act as a buffer against the existential anxiety triggered by mortality salience. In scenarios of mortality salience, anthropomorphic designs serve as an emotional regulation strategy by enhancing the interactivity between consumers and the products through the assignment of human traits to everyday items, thereby facilitating a positive emotional transformation. These designs not only reduce negative emotions driven by death anxiety but also enhance consumer preferences for anthropomorphized products through non-emotional factors such as enhanced sense of life meaning, control, psychological closeness, and belonging.

### 2.1. Mechanisms of Emotional Improvement

In contexts of mortality salience, existential anxiety increases, leading to a range of negative emotions [22]. Anthropomorphic products offer an effective means of alleviating these negative emotions by providing channels for emotional regulation [23]. Anthropomorphic design, which involves attributing human characteristics to non-human entities, offers a unique way of emotional connection, endowing items or products with human traits and emotional attributes, thereby establishing deeper user interactions [7]. This design enhances the social affinity of products, reduces the anxiety brought about by reminders of death, and fosters the generation of positive emotions [20]. Anthropomorphic features may also evoke feelings of familiarity and security, thereby promoting emotional comfort in the face of existential threats [7]. By providing a sense of social support, anthropomorphic products enhance individuals’ sense of life meaning and belonging, further mitigating the impact of death anxiety [21].

Research indicates that anthropomorphic designs significantly strengthen the emotional bonds between users and products, enhancing product appeal and user satisfaction [24,25]. As a potential mechanism of emotional regulation, particularly in facing the challenges brought by mortality salience, it provides emotional comfort and support [26]. By fostering positive social interactions and enhancing product affinity, anthropomorphic design effectively reduces the negative emotions triggered by reminders of death [20].

Furthermore, the emotional connections fostered by anthropomorphic features transcend mere aesthetic appreciation, inspiring deeper levels of emotional engagement [27]. Anthropomorphic products psychologically provide users with a virtual “companion”, especially in facing death anxiety. This companion role can offer emotional support, alleviating users’ fear and sadness through emotional resonance and psychological support, and enhancing feelings of security and well-being [23]. From a social interaction perspective, anthropomorphic products mimic human social interactions, offering users non-traditional channels of socialization, enhancing dependence on and affection for the product, thereby alleviating negative emotions triggered by mortality salience [7].

In conclusion, anthropomorphic design not only directly reduces negative emotions but also indirectly enhances personal preferences for products through an increase in positive emotions. This preference enhancement is driven by a combination of emotional comfort and social connectivity, forming a positive feedback loop of emotional response. By adding emotional value to products, anthropomorphic design becomes an effective tool for emotional regulation, particularly in handling complex emotions like death anxiety, showcasing its unique and significant role [23]. Based on this, we propose the following hypothesis:

**Hypothesis** **1.** *In contexts of mortality salience, products with anthropomorphic designs significantly reduce individuals’ negative emotions (such as fear and sadness) and enhance positive emotions (such as happiness), thereby strengthening individuals’ preferences for these products*.

### 2.2. Impact of Non-Emotional Mechanisms

Distraction tasks are a common method in mortality salience research, aimed at mitigating transient emotional responses by relegating thoughts of death to an unconscious level [28]. Beyond the direct emotional benefits, anthropomorphic design also functions through non-emotional mechanisms, enhancing users’ sense of control, belonging, life meaning, and psychological closeness.

Mortality salience often leads to a reduced sense of control; however, anthropomorphic design, through its human-like understanding and feedback mechanisms, effectively enhances users’ sense of control [29]. Specifically, intuitive interfaces and human-like feedback help individuals regain confidence in problem-solving, compensating for the control lost due to mortality salience, thereby increasing preference for anthropomorphized products [29].

By endowing products with human characteristics, anthropomorphic design promotes emotional resonance, enhancing individuals’ sense of belonging [7]. In the context of mortality salience, where social connections are threatened, anthropomorphic products become vital channels for re-establishing social ties and a sense of belonging [30]. By simulating human social behaviors, anthropomorphic design meets individuals’ strong needs for social interaction and belonging, forming a psychological buffer [7].

Mortality salience challenges individuals’ sense of life meaning, and anthropomorphic design, by deepening emotional investment, helps restore and enhance this sense of life meaning. The care and protective tendencies displayed towards anthropomorphic objects strengthen the perception of life value [21,31]. Following mortality salience, anthropomorphic products become pathways for individuals to seek life meaning, serving as essential components of psychological buffering.

Anthropomorphic design, by mimicking human social behaviors such as conversation, expressions, and gestures, establishes interactions akin to interpersonal relationships, increasing individuals’ psychological closeness [32]. Facing the pressures of mortality salience, anthropomorphic design alleviates psychological stress by enhancing feelings of emotional fulfillment and security, satisfying the individual need for psychological closeness.

Based on the above analysis, it can be inferred that even after adjusting death-related emotions to a subconscious level, products with anthropomorphic designs can still significantly enhance preferences for these products through the aforementioned non-emotional factors. This is because, although distraction tasks may temporarily shield the direct emotional impacts of death, the long-term and deeper psychological needs, such as a sense of control, belonging, life meaning, and psychological closeness, can still be effectively met through anthropomorphic design. Thus, we propose the following hypothesis:

**Hypothesis** **2.** *Even if individuals adjust death-related emotions to a subconscious level through distraction tasks, products with anthropomorphic designs can still significantly enhance preferences for these products by enhancing non-emotional factors such as a sense of life meaning, belonging, control, and psychological closeness*.

**Figure 1:**
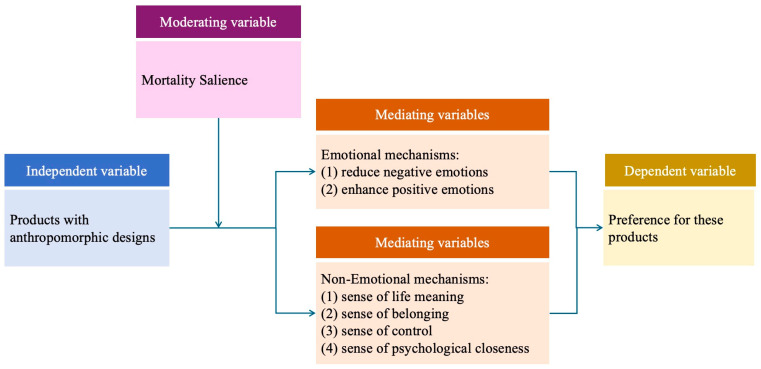
Hypothetical conceptual model.

Figure 1 summarizes the theoretical framework of this study, based on the aforementioned analysis.

## 3. Experiment 1: The Impact of Anthropomorphic Products on Individual Preferences and Emotional Changes

### 3.1. Overview of Research Design for Experiment 1

This experiment employed a 2 × 2 between-subjects design (anthropomorphism: yes/no; mortality salience: yes/no) to explore the effects of anthropomorphic and non-anthropomorphic coffee machine designs on individual product preferences and emotional states under conditions of mortality salience. Through a carefully designed experiment, we collected and analyzed participants’ subjective reports on product preferences and their emotional responses in specific scenarios.

Participants were recruited from Ningbo University in China. After excluding those who failed attention checks, a total of 160 valid samples were included (46.25% male, average age 23.26 years, SD = 3.052). These participants were randomly assigned to one of four groups, each comprising 40 individuals: “Death + Anthropomorphism”, “Death + Non-Anthropomorphism”, “Control + Anthropomorphism”, and “Control + Non-Anthropomorphism”. Each participant was offered a snack after the experiment to mitigate any potential negative emotions.

### 3.2. Experimental Materials and Methods for Experiment 1 

#### 3.2.1. Experimental Stimuli and Pretest for Experiment 1

**Figure 2:**
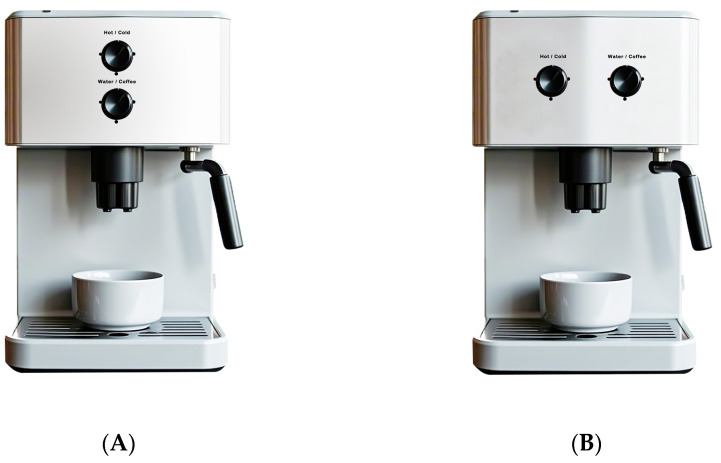
Stimuli for Experiment 1. (**A**) Non-anthropomorphic product. (**B**) Anthropomorphic design.

Leveraging anthropomorphic manipulation techniques proven effective in prior research [33,34], we designed two coffee machines as experimental stimuli, one with anthropomorphic features and the other without (see Figure 2). To ensure that participants could perceive the differences in levels of anthropomorphism, a pre-test was conducted. We recruited 30 participants offline (average age M = 25.1, SD = 1.689). Participants were first introduced to the concept of anthropomorphism, then asked to rate the extent to which the coffee machines resembled humans using a 7-point Likert scale (1 = “not at all human-like”; 7 = “very human-like”). A one-sample *t*-test was used to compare the level of anthropomorphism against the midpoint of “4”. The results showed that the non-anthropomorphic product scored significantly below 4 (M = 2.00, SD = 0.152, t(29) = −13.191, *p* < 0.001), while the anthropomorphic product scored significantly above 4 (M = 5.47, SD = 0.150, t(29) = 9.805, *p* < 0.001). To ensure no significant difference in attractiveness between the two coffee machines, participants rated the attractiveness of the designs (1 = “dislike very much”; 7 = “like very much”). The results indicated no significant difference in attractiveness between the non-anthropomorphic (M = 5.27) and anthropomorphic (M = 5.50) coffee machines (t(29) = −1.270, *p* = 0.2142).

#### 3.2.2. Procedures for Experiment 1

Following the pilot study, the main experiment proceeded as outlined below. Participants filled out basic information and underwent scenario manipulation. In the mortality salience group, participants were initially informed they were part of a survey on the psychological health levels of college students. Specifically, they were asked to answer the following two questions: (1) “Please imagine your thoughts and feelings at the moment of death and describe them in words”, and (2) “Please imagine the physical changes occurring to your body at the brink of death and after death”. In contrast, participants in the control group were told they were participating in a survey about dental health; thus, they were asked to imagine their feelings and bodily changes during tooth extraction. After completing the scenario manipulation, participants were informed they would partake in a second survey assessing the design of a coffee machine. Lastly, participants were asked to measure and report their current emotional state.

#### 3.2.3. Data Collection Methods

Study 1 utilized all scales as 7-point Likert scales, where participants reported their agreement with each item.

Regarding positive and negative emotions, the PANAS scale by Kercher (1992) [35] and Liu et al. (2024) [36] was used, including 10 items. Positive emotions included “excited”, “enthusiastic”, “alert”, “inspired”, and “determined”; negative emotions included “distressed”, “upset”, “scared”, “nervous”, and “afraid”.

Regarding preferences for product design aesthetics, we used a 2-item scale designed by Lafferty (2007) [37], asking participants, “I like the design of this coffee machine” and “I am willing to purchase a coffee machine with this design”.

### 3.3. Results of Experiment 1

#### 3.3.1. Mortality Salience Manipulation Check for Experiment 1

The independent sample *t*-test results showed that participants in the mortality salience group reported significantly more thoughts of death (M = 5.73, SD = 0.077) than the control group (M = 1.88, SD = 0.076) (t(158) = −35.413, *p* < 0.001). This confirms the effectiveness of the mortality salience manipulation.

#### 3.3.2. Main Effect Test for Experiment 1

We used independent sample *t*-tests to compare differences in preferences for anthropomorphized and non-anthropomorphized products between the mortality salience and control condition groups. Table 1 reports the statistical results.

It is evident that under mortality salience, participants’ preference for anthropomorphized products was significantly higher than for non-anthropomorphized products (Anthropomorphized: M = 5.400, SD = 0.084; Non-anthropomorphized: M = 2.763, SD = 0.096), with a significant difference (t(78) = 20.592, *p* < 0.001). This suggests that when confronted with mortality salience, participants tend to choose products with anthropomorphic features.

Conversely, in the control condition, participants’ preference for non-anthropomorphic designs was significantly higher than for anthropomorphic designs (anthropomorphized: M = 3.425, SD = 0.071; non-anthropomorphized: M = 3.875, SD = 0.076), with a similarly significant difference (t(78) = −4.292, *p* < 0.001). This indicates that in the absence of mortality salience, participants prefer non-anthropomorphic designs.

Further analysis revealed that when considering only anthropomorphized products, the preference under mortality salience was significantly higher than in the control condition (mortality group: M = 5.400; control group: M = 3.425; t(78) = 17.955, *p* < 0.001). For non-anthropomorphized products, however, the opposite trend was observed, with a significantly lower preference under mortality salience than in the control condition (mortality group: M = 2.763; control group: M = 3.875; t(78) = −8.994, *p* < 0.001).

These results support our Hypothesis 1, suggesting that consumer preference for anthropomorphized products significantly increases when confronted with mortality salience. This finding may imply the potential role of anthropomorphic design as emotional support, especially when individuals face existential threats.

#### 3.3.3. Emotional Differences Between Anthropomorphic and Non-Anthropomorphic Designs

##### Regression Analysis of Emotional Impact

In accordance with Hypothesis 1’s theoretical expectations, the preference for anthropomorphized coffee machine designs under mortality salience was driven by emotions. Hence, we employed a multivariate linear regression model to further explore the relationship between consumer preferences and emotions. In this regression model, participants’ reported scores of positive and negative emotions served as the dependent variables, while the presence of anthropomorphic features in the coffee machine design, whether the participant was under mortality salience, and their interaction terms served as explanatory variables. Factors such as age and gender were controlled to minimize potential confounding effects. Table 2 provides a detailed list of the regression analysis results.

The regression results from Model 1 in Table 2 indicate that under mortality salience, participants exhibited a significant decrease in positive emotions (β = −0.472, *p* < 0.001). However, the presence of anthropomorphic design did not significantly affect participants’ positive emotions (β = 0.143, *p* > 0.05). Additionally, the interaction term between “anthropomorphic design” and “mortality salience” also did not significantly impact participants’ positive emotions (β = −0.055, *p* > 0.05). This suggests that in the current study, anthropomorphic design did not significantly alter the impact of mortality salience on participants’ positive emotions. In other words, regardless of whether participants engaged with anthropomorphized product designs, their emotional response to mortality salience was statistically similar.

The regression results from Model 2 in Table 2 show that mortality salience significantly increased participants’ negative emotions (β = 1.995, *p* < 0.001), while the presence of anthropomorphic design significantly reduced their negative emotions (β = −0.226, *p* < 0.001). This indicates that individuals indeed experience heightened negative emotions when confronted with mortality-related scenarios, and designs featuring anthropomorphic characteristics can mitigate these negative emotions to some extent. Importantly, the interaction between “anthropomorphic design” and “mortality salience” also had a significant negative impact on participants’ negative emotions (β = −0.811, *p* < 0.001). This suggests that under mortality salience, anthropomorphic product designs not only reduce negative emotions but do so more significantly than in non-mortality salient conditions. In other words, when facing mortality-related stress or anxiety, anthropomorphic product designs may serve as an emotional “safe harbor”, effectively alleviating negative emotions.

Overall, the regression results from Table 2 provide strong support for Hypothesis 1. They demonstrate that although anthropomorphic design does not significantly enhance positive emotions under mortality salience, it does significantly reduce negative emotions and may thereby increase individual preferences for these products. These findings have significant psychological implications for understanding consumer behavior when confronted with the finiteness of life and provide empirical evidence for the role of emotional factors in product design.

##### Further Analysis of Negative Emotions

Previous analysis indicated that while anthropomorphic design did not significantly boost positive emotions under mortality salience, it did significantly reduce negative emotions. In this section, we delve deeper into the impact of anthropomorphic design on various types of negative emotions under mortality salience. We use data from the mortality salience group, with participants’ reported levels of “distressed”, “upset”, “scared”, “nervous”, and “afraid” serving as dependent variables. “Anthropomorphism” is included as the core explanatory variable, with gender and age controlled to eliminate potential confounding effects. Table 3 presents detailed regression results.

The regression results from Table 3 show that “anthropomorphism” has a negative coefficient for all negative emotion indicators, and these coefficients are statistically significant at the 0.1% level. Specifically, “anthropomorphism” has a regression coefficient of −0.986 for “distressed”, −0.559 for “upset”, −1.417 for “scared”, −0.736 for “nervous”, and −1.480 for “afraid”.

These results statistically confirm our hypothesis that, in a mortality salience context, anthropomorphic design significantly reduces individuals’ negative emotions. More specifically, anthropomorphic design plays a positive role in reducing participants’ distress, unease, fear, nervousness, and fright. This further corroborates the expectations of Hypothesis 1, suggesting that in scenarios related to death, anthropomorphic design may act as an effective emotional regulation strategy, helping individuals mitigate the stress of negative emotions.

### 3.4. Discussion on Experiment 1

#### 3.4.1. Results and Analysis of Experiment 1

This study aimed to explore the effects of anthropomorphic design on individual product preferences and emotional states in a mortality salience context. Through a 2 × 2 between-subjects design, we successfully demonstrated that in mortality salience contexts, coffee machines with anthropomorphic designs were preferred over non-anthropomorphic designs and significantly reduced participants’ negative emotion levels. The results showed that under mortality salience conditions, participants distinctly favored anthropomorphic products over non-anthropomorphic ones, while the opposite trend appeared under control conditions. Moreover, multivariate regression analysis further confirmed the effectiveness of anthropomorphic design in alleviating negative emotions, especially evident in the emotional dimensions of “distressed”, “upset”, “scared”, “nervous”, and “afraid”. These findings provide strong evidence in support of Hypothesis 1, indicating that anthropomorphic design, as an emotional regulation tool, has potential applications in existential threat scenarios.

#### 3.4.2. Limitations and Directions for Improvement of Experiment 1

Despite providing valuable preliminary data, Experiment 1 has several limitations that future research needs to address. Firstly, the choice of a functional product—coffee machines—may not favor the realization of anthropomorphic design intentions due to its inherent industrial properties. In response, subsequent studies plan to use hedonic products as new experimental stimuli to explore the effects of anthropomorphic design across different product categories and its consistency in influencing individual emotional responses.

Secondly, although Experiment 1 preliminarily verified the mediating role of negative emotions between anthropomorphic design and product preference, to ensure the robustness of these conclusions, future research intends to use other types of emotional scales to further validate the mechanisms by which anthropomorphic design modulates individual emotional states.

Lastly, the control condition in this experiment involved a “toothache scenario”, which may indirectly trigger negative emotions in participants, potentially confounding the results. Therefore, it is recommended that subsequent studies design more neutral control scenarios to eliminate possible confounding factors and ensure the consistency and reliability of experimental conditions and outcomes.

In conclusion, Experiment 1 provides a preliminary empirical basis for the application of anthropomorphic design in mortality salience contexts, but further research is needed to overcome existing limitations and deepen our understanding of this phenomenon.

## 4. Experiment 2: Further Testing Emotional Changes Using Different Experimental Stimuli

### 4.1. Overview of Research Design for Experiment 2

Experiment 2 continues to follow a 2 (anthropomorphism: yes vs. no) × 2 (emotional context: mortality salience vs. neutral) between-subjects design. To validate the robustness of Hypothesis 1 and to further explore the underlying emotional regulation mechanisms, we changed the experimental stimuli to hedonic products—light sticks; used different emotional scales; and employed a neutral priming scenario in the control group.

We once again recruited 160 valid participants at Ningbo University in China, of whom 50.63% were male, with an average age of 23.17 years (SD = 2.382). All participants were randomly and evenly assigned to one of four experimental groups: “Mortality Salience + Anthropomorphized”, “Mortality Salience + Non-Anthropomorphized”, “Control + Anthropomorphized”, and “Control + Non-Anthropomorphized”. Each participant received a snack as compensation after completing the experiment.

### 4.2. Experimental Materials and Methods for Experiment 2

#### 4.2.1. Selection Criteria for Anthropomorphized and Non-Anthropomorphized Products

**Figure 3:**
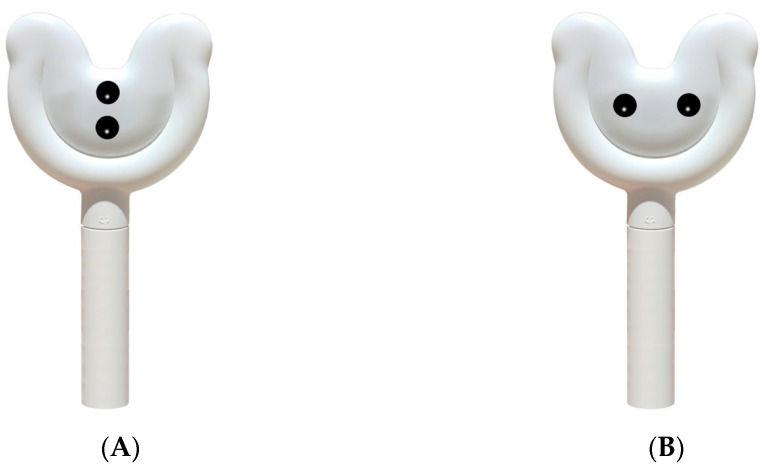
Stimuli for Experiment 2. (**A**) Non-anthropomorphic product. (**B**) Anthropomorphic design.

We designed two light sticks as the stimuli for this experiment, as shown in Figure 3. To verify the effectiveness and perceptibility of the stimuli in Experiment 2, we conducted a pretest. We invited 30 students from Ningbo University to participate in this pretest. First, we thoroughly explained the definitions of hedonistic and functional products to ensure participants had a clear understanding of these two product types. Then, we asked participants to evaluate the hedonistic characteristics of the light sticks on a 7-point scale, where 1 represented “strongly disagree” and 7 represented “strongly agree”. To verify the anthropomorphic design features of the light sticks, we also asked participants to rate the level of anthropomorphism in the design of the sticks, ranging from 1 (“not human-like at all”) to 7 (“very human-like”). Additionally, to control for other potential variables, we assessed the aesthetic appeal of the two light sticks.

The results indicated that participants significantly perceived the light sticks as hedonistic products (M = 5.20, SD = 0.242). A *t*-test showed that this perception was significantly higher than the midpoint (t(29) = 4.966, *p* < 0.001), confirming the effectiveness of our experimental stimuli regarding hedonistic characteristics. In the evaluation of anthropomorphic levels, participants found the anthropomorphized light stick significantly more human-like than the non-anthropomorphized stick (M_anthrop = 5.43, M_non-anthrop = 2.20). The *t*-test results indicated that this difference was extremely significant (t(29) = 11.981, *p* < 0.001), suggesting that participants could clearly identify the anthropomorphic design features on the light sticks. Moreover, there was no significant difference in the aesthetic appeal scores between the two designs (M_anthrop = 3.03, M_non-anthrop = 3.00; t(29) = 0.120, *p* = 0.9052). This outcome indicated that the anthropomorphic design did not significantly affect aesthetic appeal, thereby eliminating the possibility of aesthetic appeal as a confounding variable.

#### 4.2.2. Procedures for Experiment 2

First, participants were randomly and evenly distributed among the four experimental groups. The mortality salience group received the same death salience materials as in Experiment 1, while the control group received neutral priming materials, being asked to recall scenes from a recent TV show they watched. Subsequently, participants filled out an emotional state questionnaire and engaged in a survey about the product design, reporting their preference for and willingness to purchase the light sticks. Finally, they reported their emotional state again to assess changes. In Experiment 2, we used the six basic emotional scales by An et al. (2017) [38] to evaluate participants’ emotional levels.

### 4.3. Results of Experiment 2

#### 4.3.1. Mortality Salience Manipulation Check for Experiment 2

To verify the effectiveness of the mortality salience manipulation, we conducted an independent sample *t*-test. The results showed that the mortality salience group reported significantly higher thoughts of death (M = 5.425, SD = 0.073) compared to the control group (M = 2.400, SD = 0.072) (t(158) = 29.435, *p* < 0.001). This confirmed the effectiveness of the mortality salience manipulation.

#### 4.3.2. Main Effect Test for Experiment 2

We examined participants’ preferences for the experimental stimuli under different group conditions using independent sample *t*-tests, as shown in Table 4.

As can be seen from Table 4, in the mortality salience context, participants showed a significantly higher preference for anthropomorphized products (M = 4.200, SD = 0.089) compared to non-anthropomorphized products (M = 3.225, SD = 0.074) (t(78) = 8.429, *p* < 0.001). In the control context, there was no significant difference between the preference for non-anthropomorphized designs (M = 3.400, SD = 0.082) and anthropomorphized designs (M = 3.525, SD = 0.067) (t(78)= 1.178, *p* = 0.2425). Analyzing all data for anthropomorphized products, the preference in the mortality group (M = 4.200) was significantly higher than in the control group (M = 3.525) (t(78) = 6.057, *p* < 0.001). In addition, for all non-anthropomorphized products, there was no significant difference in preference between the mortality group (M = 3.225) and the control group (M = 3.400) (t(78) = −1.583, *p* = 0.1176).

The results from Table 4 further support Hypothesis 1, demonstrating that in contexts of mortality salience, consumers’ preferences for products with anthropomorphic designs significantly increase. These findings not only validate the robustness of the conclusions from Experiment 1 but also enhance the external validity through different experimental stimuli and emotional scales.

#### 4.3.3. Analysis of Emotional Changes

**Figure 4:**
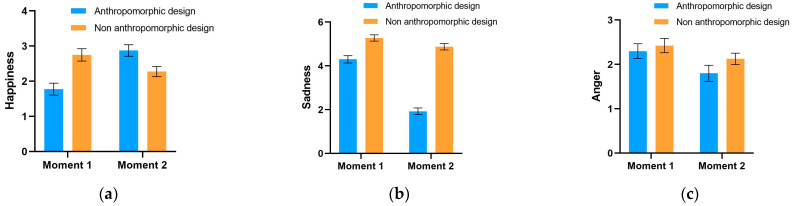

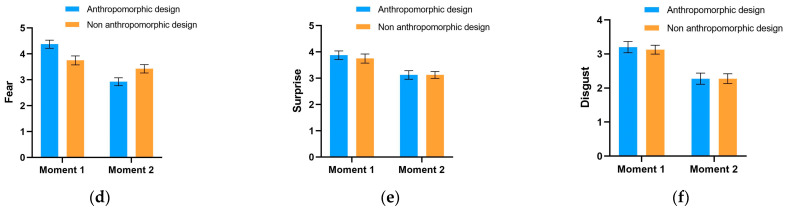
Emotional changes in the mortality salience group before and after viewing (non-)anthropomorphic products. Figures (**a**–**f**) represent the results for Happiness, Sadness, Anger, Fear, Surprise, and Disgust, respectively.

In this section, we analyzed the differential impacts of anthropomorphic design on participants’ emotions under conditions of mortality salience and control. As shown in Figure 4, we recorded the emotional changes of participants before and after evaluating anthropomorphized and non-anthropomorphized designs. Emotional changes were measured through self-assessment of six basic emotions (“happiness”, “sadness”, “anger”, “fear”, “surprise”, and “disgust”), with the y-axis representing the intensity of emotions. “Moment 1” refers to the time after the mortality salience manipulation but before exposure to the experimental stimuli, and “Moment 2” refers to the time after participants evaluated the experimental stimuli.

From Figure 4, in the anthropomorphized design group, happiness significantly increased from “Moment 1” to “Moment 2”, and the other five negative basic emotions significantly decreased. However, this alone does not prove the emotional effects produced by anthropomorphic design, as a similar trend in emotional changes was also observed in the non-anthropomorphized group. Therefore, the reported emotional changes at Moments 1 and 2 could also be attributed to other factors, such as the natural alleviation of tension over time. Hence, to accurately assess the impact of anthropomorphic design, it is necessary to use the non-anthropomorphized group as a baseline and employ a difference-in-differences (DID) approach for this test.

**Figure 5:**
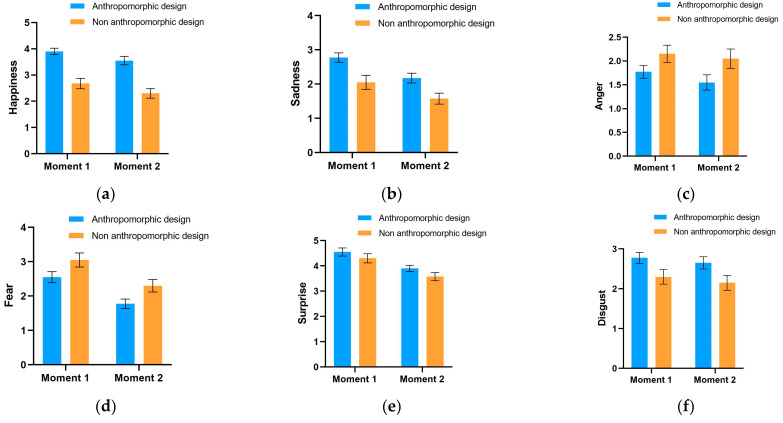
Emotional changes in the control group before and after viewing (non-)anthropomorphic products. Figures (**a**–**f**) represent the results for Happiness, Sadness, Anger, Fear, Surprise, and Disgust, respectively.

Figure 5 records the emotional changes of participants in the control group using the same method as Figure 4. Comparing the reported emotional changes before and after evaluating the experimental stimuli for both anthropomorphized and non-anthropomorphized groups, a decrease in all six basic emotions was observed. Therefore, to determine whether these emotional changes were caused by anthropomorphic design, a DID test is similarly required.

To quantitatively test the impact of anthropomorphic design on emotions, we employed the difference-in-differences (DID) approach, comparing the emotional changes between the anthropomorphized group (treatment group) and the non-anthropomorphized group (control group) under both mortality salience and non-mortality salience conditions. We used the six basic emotions (“happiness”, “sadness”, “anger”, “fear”, “surprise”, and “disgust”) as dependent variables, “exposure to anthropomorphic design” as the treatment variable, and the time before and after exposure as the time variable for DID analysis. Additionally, we controlled for individual fixed effects in the regression model and used heteroscedasticity-robust standard errors for statistical inference, ensuring the accuracy and robustness of the results. 

Table 5 reports the DID regression results for the treatment group. The DID regression results presented in this table show that in the models using the six basic emotions as dependent variables, the interaction term regression coefficient for happiness is significantly positive (1.575, *p* < 0.001). This indicates that under conditions of mortality salience, participants exposed to anthropomorphic designs experienced a significant increase in happiness after evaluating the experimental stimuli.

Furthermore, the interaction term regression coefficients for sadness and fear are −1.975 and −1.125, respectively, both reaching statistical significance (*p* < 0.001). This further suggests that anthropomorphic designs can significantly reduce sadness and fear emotions in contexts of mortality salience.

However, the interaction term regression coefficients for anger, surprise, and disgust are not significant, indicating that the effects of anthropomorphic design on these emotions are not apparent.

Table 6 reports the DID regression results for the control group. In stark contrast to the mortality salience group, the interaction term regression coefficients for all six basic emotions as dependent variables are not significant. This implies that under non-mortality salience conditions, anthropomorphic design does not significantly affect participants’ emotions.

These regression results support our hypothesis that under conditions of mortality salience, consumers significantly increase their preference for products with anthropomorphic designs, accompanied by positive emotional changes. Specifically, under mortality salience conditions, anthropomorphic design significantly enhances participants’ happiness and reduces their sadness and fear, further validating the positive role of anthropomorphic design in emotional regulation.

However, it is noteworthy that in the control group, the impact of anthropomorphic design on emotions is not significant. This may be because, in the absence of mortality salience, consumers are less sensitive to product design or their emotional responses are not as intense. This further highlights the crucial role of mortality salience context in eliciting consumer emotional responses and preference changes.

### 4.4. Discussion on Experiment 2

This study, through Experiment 2, further validates the robustness of Hypothesis 1 and delves deeper into the impact of anthropomorphic design on individual emotions and product preferences under mortality salience conditions. The results show that under conditions of mortality salience, participants exhibit a higher preference for anthropomorphized light sticks, consistent with the findings from Experiment 1, thereby enhancing the external validity of our research conclusions. More importantly, the emotional change analysis using the difference-in-differences (DID) method reveals the positive role of anthropomorphic design in emotional regulation: under mortality salience conditions, anthropomorphic design not only significantly enhances participants’ happiness but also effectively reduces their sadness and fear.

The results of Experiment 2 suggest that anthropomorphic design serves as an effective emotional regulation strategy, significantly alleviating negative emotions under conditions of mortality salience and enhancing positive emotions. These findings not only enrich the application scenarios of terror management theory (TMT) but also provide practical guidance for product design—integrating anthropomorphic elements into hedonic products may enhance their appeal in specific contexts.

However, Experiment 2 also finds that under non-mortality salience conditions, the impact of anthropomorphic design on participants’ emotions and product preferences is not significant. This result suggests that the effectiveness of anthropomorphic design may be contingent on contextual factors, particularly in settings without intense emotional arousal, where its effects may not be evident.

In conclusion, the results of Experiment 2 support the view of anthropomorphic design as an effective emotional regulation tool, especially in contexts facing life threats. These findings not only deepen our understanding of how anthropomorphic design influences consumer emotions and product preferences but also provide new perspectives for future research.

## 5. Experiment 3: Exploring the Durability of Emotional Regulation and the Impact of Non-Emotional Mechanisms

### 5.1. Overview of Research Design for Experiment 3

Experiment 3 aims to verify whether consumers’ preferences for anthropomorphized products remain stable over time, even as the emotional effects diminish. This experiment also explores whether such preferences can be maintained through non-emotional mechanisms after a distractor task. Continuing the 2 × 2 factorial design (anthropomorphism: yes vs. no; emotional context: mortality salience vs. neutral) used in prior studies, Experiment 3 introduces a distractor task to probe the non-emotional impacts of mortality salience.

We recruited 160 valid participants for this experiment, 38.75% of whom were male, with an average age of 22.61 years. These participants were randomly assigned to one of four experimental groups and received a snack as compensation after the experiment.

### 5.2. Experimental Methods and Materials for Experiment 3

#### 5.2.1. Procedures for Experiment 3

Participants began by completing a personal information form. They were then exposed to the designated emotional context based on their group assignment. To serve the purpose of distraction, participants were asked to participate in a task purportedly assessing the cognitive agility of college students. During this task, participants were required to solve a series of mathematical problems (e.g., “(56 − 38)*2 − 11”) within a set time limit to ensure their focus was solely on the task at hand, thereby minimizing the influence of the prior emotional context on subsequent behavior. Following this, participants were asked to rate their preference for specific anthropomorphic or non-anthropomorphic design light sticks. Finally, to gain a comprehensive understanding of the participants’ emotional states and the potential influence on product preference, researchers asked participants to complete several scales assessing dimensions of psychological state, including sense of life meaning, sense of control, psychological closeness, and sense of belonging.

#### 5.2.2. Experimental Materials and Scales

The stimuli used in Experiment 3 were consistent with those used in Experiment 2, thereby omitting the pre-experimental part. The methods of situational manipulation for the mortality salience and control groups, as well as the product preference rating scales, remained the same as in the previous phase. Furthermore, assessments of sense of control, belonging, life meaning, and psychological closeness were conducted using revised scales (see Table 7). Through these steps, this study systematically examines whether anthropomorphic product designs can still enhance consumer preference by boosting perceptions in non-affective aspects after a distractor task.

### 5.3. Results of Experiment 3

#### 5.3.1. Mortality Salience Manipulation Check for Experiment 3

To verify the effectiveness of the mortality salience manipulation, we conducted an independent samples *t*-test. The results indicated that participants in the mortality salience group reported significantly more death-related thoughts than those in the control group (mortality salience group: M = 5.338, SD = 0.119; control group: M = 2.088, SD = 0.127) (t(158) = 18.633, *p* < 0.001). This confirms that our manipulation successfully elicited considerations of death among the participants.

#### 5.3.2. Participants’ Preferences for Different Light Sticks

Table 8 presents the preferences of participants for the light sticks across the experimental groups. The findings reveal that under mortality salience conditions, participants exhibited a higher preference for products with anthropomorphic designs (M = 5.375, SD = 0.106) compared to those with non-anthropomorphic designs (M = 3.525, SD = 0.243), with a significant difference (t(78) = 6.988, *p* < 0.001). In contrast, in the neutral condition, there was no significant difference in preference between the two types of products (anthropomorphic vs. non-anthropomorphic; M = 3.700 vs. M = 3.500, t(78) = 0.528, *p* = 0.5995).

Further analysis indicated that within the anthropomorphic product category, the ratings of the mortality salience group were significantly higher than those of the neutral group (M = 5.375 vs. M = 3.700, t(78) = 5.947, *p* < 0.001). However, there was no significant difference in preference ratings between the groups for non-anthropomorphic products (M = 3.525 vs. M = 3.500, t(78) = −0.068, *p* = 0.9458).

The results of Table 8 supported Hypothesis 2, demonstrating that even when emotional responses are reduced to a subconscious level by a distractor task, products with anthropomorphic designs can still enhance consumer preferences through non-affective factors. Furthermore, these effects were not significantly altered in neutral contexts, consistent with previous research suggesting that the appeal of anthropomorphic designs may not be as pronounced under normal conditions.

After exposure to mortality salience, the anthropomorphic designs notably increased product appeal, potentially due to the added psychological value provided by elements that enhance the sense of life meaning or offer a sense of social connection, thereby alleviating death anxiety.

#### 5.3.3. Results of the Mediation Effect Analysis

**Figure 6:**
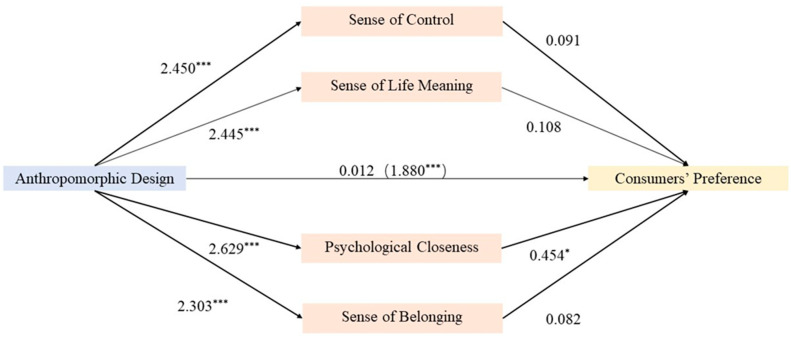
Mediation effect analysis of non-emotional factors. Note: * *p* < 0.05; *** *p* < 0.001.

To further verify Hypothesis 2—those products with anthropomorphic designs can increase consumer preferences through enhanced non-affective factors such as sense of life meaning, belonging, control, and psychological closeness—we conducted a mediation effect test based on the data from the anthropomorphism group. The results of the mediation effect analysis are summarized in Figure 6.

Initially, we performed a regression analysis with preference for the light sticks as the dependent variable and the presence of anthropomorphic features as the independent variable. The results showed a significant positive coefficient for “anthropomorphic features” (β = 1.880, *p* < 0.001), indicating that anthropomorphic designs indeed increased participants’ preferences for the light sticks.

Subsequently, we explored whether anthropomorphic features indirectly influenced preferences by enhancing non-affective factors. We conducted separate regression analyses with sense of life meaning, belonging, control, and psychological closeness as dependent variables. The results indicated that anthropomorphic features had a significant positive impact on all four non-affective factors (β ranging from 2.303 to 2.629, *p* < 0.001), suggesting that products with anthropomorphic designs significantly enhance participants’ perceptions of these non-affective factors.

Finally, to test whether these non-affective factors mediated the relationship between anthropomorphic design and product preference, we included all four mediators in the regression model. The direct effect of “anthropomorphism” became non-significant (β = 0.012, *p* = 0.978), indicating a complete mediation effect. Among the mediators, only psychological closeness had a significant positive regression coefficient (β = 0.454, *p* < 0.05), suggesting that psychological closeness primarily mediated this process. The other three mediators—sense of life meaning, belonging, and control—although significantly associated with anthropomorphic design, did not show significant mediation effects in this model, possibly due to high collinearity with psychological closeness or because their effects were overshadowed by psychological closeness.

The mediation analysis supports Hypothesis 2, suggesting that even when emotional effects are minimized by a distractor task, anthropomorphic designs can still enhance consumer preferences through these non-affective factors, particularly psychological closeness. Psychological closeness, as a significant mediator, indicates that anthropomorphic designs may enhance consumers’ positive attitudes and preferences towards products by providing a sense of psychological proximity and connection. This psychological closeness likely stems from the “human-like” characteristics endowed by anthropomorphic designs, allowing consumers to more easily form emotional connections and identification with the products.

### 5.4. Discussion on Experiment 3

The results of Experiment 3 confirm Hypothesis 2, demonstrating that consumer preferences for products with anthropomorphic designs are significantly enhanced even when mortality salience emotions are reduced to a subconscious level by a distractor task. Further mediation effect analysis indicated that psychological closeness fully mediates the relationship between anthropomorphic design and product preference, while sense of life meaning, belonging, and control, although related to anthropomorphic design, did not show significant mediation effects.

These findings emphasize the importance of anthropomorphic designs in mitigating the negative emotions associated with mortality salience and reveal the mechanism by which they enhance product preferences through the enhancement of psychological closeness. Not only do these findings deepen our understanding of the utility of anthropomorphic designs, but they also provide a theoretical basis for marketing strategies.

## 6. Conclusions and Discussion

### 6.1. Summary of Research Findings

This study investigated the impact of anthropomorphic product design on consumer preferences and emotions under conditions of mortality salience, aiming to validate whether anthropomorphic design enhances consumer preference through emotional and non-emotional mechanisms. Hypotheses 1 and 2 were tested through three experiments. Results from Experiments 1 and 2 supported Hypothesis 1, demonstrating that under mortality salience, consumers exhibited a higher preference for anthropomorphic products, accompanied by positive emotional changes, including significant increases in happiness and notable reductions in sadness and fear. Experiment 3 further corroborated Hypothesis 2, indicating that even when emotional responses were minimized to a subconscious level through distraction tasks, anthropomorphic design continued to significantly enhance consumer preference through non-emotional factors such as sense of life meaning, belongingness, control, and psychological closeness, with psychological closeness playing a pivotal mediating role. These findings suggest that under mortality salience, anthropomorphic design not only acts as an emotional regulation tool, mitigating individuals’ negative emotional stress, but also enhances psychological connection with the product by endowing it with human-like traits, thereby increasing consumer preference.

### 6.2. Theoretical and Practical Implications

#### 6.2.1. Theoretical Implications

This research makes innovative contributions to the intersection of terror management theory (TMT), emotion regulation theory, and consumer behavior, filling a gap in the existing literature. Traditional TMT studies have predominantly focused on the effects of mortality salience on social behavior, cognition, or attitudes, with less attention given to the regulatory role of product design elements on consumer emotions and preferences. By empirically validating how anthropomorphic design serves as a unique emotional regulation strategy under mortality salience, this study extends the theoretical boundaries of TMT. It demonstrates that anthropomorphic design can act as an emotional buffer in the presence of existential threats, not only alleviating individuals’ negative emotions but also stimulating positive consumer preferences toward the product.

Furthermore, this study delves into the non-emotional impacts of anthropomorphic design, showcasing its positive roles in aspects such as control, psychological closeness, and belongingness. The discovery of these non-emotional regulation mechanisms provides new insights into emotion regulation theory, indicating that emotion regulation is not solely reliant on direct emotional responses but can also profoundly influence consumer behavior through stable psychological connections and feelings of control. By revealing the mediating role of psychological closeness between anthropomorphic design and consumer preference, this research provides empirical evidence for the multidimensional utility of anthropomorphic design, expanding its applicability in consumer behavior studies. Additionally, the external intervention pathways proposed for anthropomorphic design offer new directions for future research on emotion regulation and consumer behavior.

#### 6.2.2. Practical Implications

From a practical application standpoint, the findings of this study provide clear guidance for product design and marketing, especially when targeting specific groups often exposed to high mortality salience. Firstly, the research indicates that in high-stress contexts, products with anthropomorphic designs can alleviate death anxiety and enhance psychological well-being by fostering psychological connections and a sense of belonging. For professional groups such as medical personnel, firefighters, and military personnel, introducing products with anthropomorphic designs in their work environments or rest areas—such as equipment and decorations with friendly appearances or human-like features—can effectively support their mental health, helping them cope with long-term stress and increasing job satisfaction.

Secondly, in terms of product design, brands can incorporate anthropomorphic elements into appropriate consumer contexts to enhance the emotional appeal and competitive edge of their products. For instance, in the design of household appliances and personal care products closely related to daily life, designers can integrate features like smiling appearances, friendly expressions, or interactive anthropomorphic elements to evoke consumers’ feelings of psychological closeness and belonging. In marketing strategies, brands could specifically promote products with anthropomorphic designs to consumer groups under significant stress (such as professionals and emergency personnel), enhancing the emotional value and market appeal of the brand by strengthening the psychological connection between the product and the users. This strategy provides a viable path for brands to create emotional differentiation advantages in competitive markets.

In summary, this study fills a theoretical gap at the intersection of terror management theory (TMT), emotion regulation theory, and consumer behavior, providing new directions for future research. Practically, it clarifies the real-world application value of anthropomorphic design in specific consumer contexts, helping designers and brands optimize emotional support and market positioning strategies to achieve both social care and commercial value in product design.

### 6.3. Research Limitations and Future Directions

Despite valuable findings, this study has limitations that warrant further improvement and exploration in future research. Firstly, the sample primarily consisted of university students from the eastern region of China, whose age, cultural background, and life experiences are relatively homogenous, possibly limiting the generalizability of the results. Future research could validate these findings across more diverse samples, including consumers of different ages, cultural backgrounds, and socioeconomic statuses, to enhance the external validity of the results.

Secondly, the mortality salience manipulation in the experimental settings relied on self-reports, without incorporating physiological or behavioral data, which might not fully capture the emotional changes of consumers in real situations. Future studies could include neurophysiological or behavioral response indicators (such as skin conductance response, heart rate variability, etc.) to more accurately monitor emotional changes and further validate the effectiveness of anthropomorphic design in addressing existential anxiety.

Lastly, the anthropomorphic design in the study was implemented solely through surface features (such as appearance); future research could explore more interactive and dynamic anthropomorphic designs, such as enhancing consumers’ emotional experiences through voice or tactile feedback.

## Figures and Tables

**Table 1:** Participants’ preferences for experimental stimuli across different groups.

Condition	Anthropomorphic Coffee Machine	Non-Anthropomorphic Coffee Machine	N	t
Mortality	5.400 ± 0.171	2.763 ± 0.195	80	20.591 ***
Control	3.425 ± 0.143	3.875 ± 0.157	80	−4.292 ***
N	80	80		
t	17.955 ***	−8.994 ***		

Note: *** *p* < 0.001.

**Table 2:** Regression results on the impact of anthropomorphic design on emotions.

	Model 1	Model 2
Variable	Positive Emotions	Negative Emotions
Anthropomorphism	0.143	−0.226 ***
	(0.090)	(0.085)
Mortality salience	−0.472 ***	1.995 ***
	(0.121)	(0.106)
Anthropomorphism × mortality salience	−0.055	−0.811 ***
	(0.159)	(0.149)
Age	−0.00005	−0.006
	(0.014)	(0.012)
Male	0.234	−0.145
	(0.080)	(0.076)
Intercept	2.180 ***	2.014 ***
	(0.335)	(0.301)
N	160	160
F	10.890 ***	105.620 ***

Note: Robust standard errors are in parentheses. The *** indicates significance at the 0.1% levels.

**Table 3:** Emotional responses in mortality salience condition.

	Model 1	Model 2	Model 3	Model 4	Model 5
Variable	Distressed	Upset	Scared	Nervous	Afraid
Anthropomorphism	−0.986 ***	−0.559 ***	−1.417 ***	−0.736 ***	−1.480 ***
	(0.160)	(0.136)	(0.126)	(0.123)	(0.147)
Age	−0.002	−0.007	0.009	−0.005	−0.012
	(0.026)	(0.022)	(0.020)	(0.020)	(0.024)
Male	−0.144	−0.093	−0.262 *	−0.124	−0.023
	(0.160)	(0.136)	(0.126)	(0.122)	(0.147)
Intercept	2.712 ***	3.492 ***	3.511 ***	4.674 ***	5.289 ***
	(0.623)	(0.529)	(0.493)	(0.478)	(0.575)
N	80	80	80	80	80
R-sq	0.335	0.184	0.627	0.323	0.571

Note: Robust standard errors are in parentheses. The * and *** indicate significance at the 5% and 0.1% levels.

**Table 4:** Preferences for experimental stimuli by group.

	Anthropomorphic Light Stick	Non-Anthropomorphic Light Stick	N	t
Mortality	4.200 ± 1.803	3.225 ± 0.149	80	8.429 ***
Control	3.525 ± 0.135	3.400 ± 0.167	80	1.178
N	80	80		
t	6.057 ***	−1.583		

Note: *** *p* < 0.001.

**Table 5:** Difference-in-differences analysis results of emotional changes after mortality salience.

	Model 1	Model 2	Model 3	Model 4	Model 5	Model 6
Variable	Happiness	Sadness	Anger	Fear	Surprise	Disgust
DID	1.575 ***	−1.975 ***	−0.200	−1.125 ***	−0.125	−0.0750
	(0.093)	(0.110)	(0.109)	(0.109)	(0.104)	(0.071)
Intercept	1.950 ***	6.175 ***	2.763 ***	4.950 ***	4.500 ***	4.050 ***
	(0.070)	(0.083)	(0.081)	(0.082)	(0.078)	(0.053)
N	160	160	160	160	160	160
R-sq	0.809	0.924	0.425	0.825	0.693	0.889

Note: Robust standard errors are in parentheses. *** *p* < 0.001.

**Table 6:** Difference-in-differences analysis results of emotional changes in non-mortality salience group.

	Model 1	Model 2	Model 3	Model 4	Model 5	Model 6
Variable	Happiness	Sadness	Anger	Fear	Surprise	Disgust
DID	0.025	−0.125	−0.125	−0.025	0.075	0.025
	(0.109)	(0.112)	(0.082)	(0.096)	(0.105)	(0.078)
Intercept	3.650 ***	2.950 ***	2.125 ***	3.563 ***	5.112 ***	2.675 ***
	(0.082)	(0.084)	(0.062)	(0.072)	(0.078)	(0.058)
N	160	160	160	160	160	160
R-sq	0.363	0.545	0.187	0.763	0.690	0.139

Note: Robust standard errors are in parentheses. *** *p* < 0.001.

**Table 7:** Adapted scales for sense of control, sense of belonging, sense of meaning in life, and psychological closeness.

Scale	Source	Items	Reliability
Sense of control	Fritsche et al. [39]	1. This product alleviates my helplessness; 2. This product eases my feelings of powerlessness; 3. This product enhances my sense of control; 4. This product makes me feel capable of changing things.	0.929
Sense of belonging	Wesselmann et al. [40]	1. This product makes me feel accepted; 2. This product enhances my interaction with it.	0.713
Sense of meaning in life	Routledge and Juhl [41]	1. This product makes me feel that life is worth living; 2. Having this product makes me feel that my existence is meaningful; 3. Having this product clarifies my life goals; 4. Owning it helps to enhance my ability to find meaning in life.	0.944
Psychological closeness	Ha and Perks [42]; Ren et al. [43]	1. This light stick gives me a sense of kindness and friendliness; 2. I feel a close connection with this light stick.	0.814

**Table 8:** Preferences for (non-)anthropomorphic products in mortality and control conditions.

	Anthropomorphic Light Stick	Non-Anthropomorphic Light Stick	N	t
Mortality	5.375 ± 0.213	3.525 ± 0.491	80	6.988 ***
Control	3.700 ± 0.528	3.500 ± 0.557	80	0.527
N	80	80		
t	5.947 ***	0.068		

Note: *** *p* < 0.001.

## Data Availability

The raw data supporting the conclusions of this article will be made available by the authors on request.
